# Supplementary material for: B7-H3 in Medulloblastoma-Derived Exosomes; A Novel Tumorigenic Role
Source: Int J Mol Sci. 2020 Sep 25;21(19):7050. doi: 10.3390/ijms21197050 (PMC7583814; doi:10.3390/ijms21197050)
Supplement: Supplementary file 1 [file ijms-21-07050-s001.pdf]

Supplementary Figure 1: B7-H3 levels in MB cells. A. Western blot analysis of D283 control and B7-H3_OE cell lysates to check the B7-H3 levels. B. Western blot showing levels of B7-H3 in different MB cell lysates. Actin was used as a loading control.

Supplementary Figure 2: F-actin and Calcein AM stains. A. The negative control to confirm non- specific Calcein AM staining was done using SFM (processed in the same way as exosome extraction) overlayed on D458 cells followed by F-actin staining. B. D458 cells incubated with Calcein AM reagent (positive control).

Supplementary Figure 3: Original Immunoblots. Original full-length immunoblot blots that correspond to Figure 3C in the manuscript. Actin was used as a loading control.

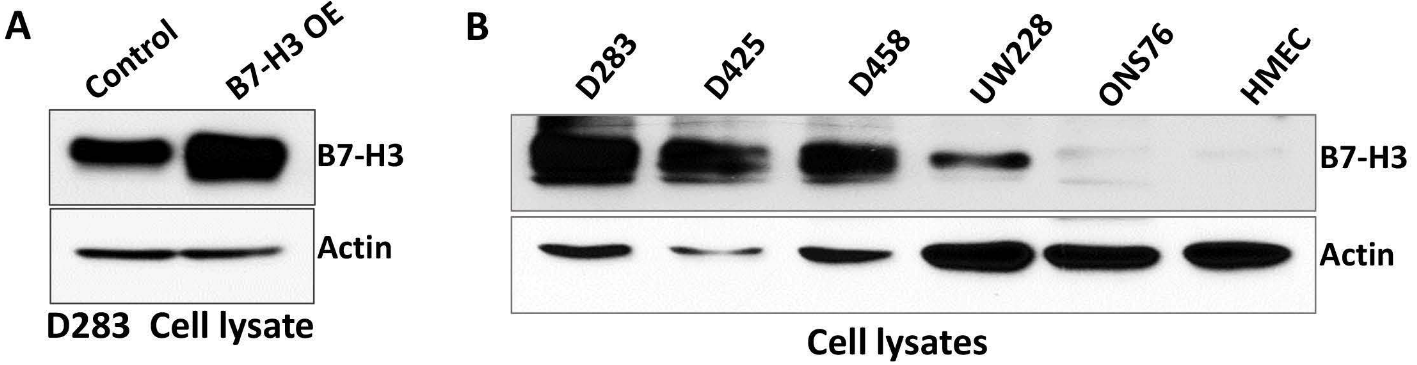

A
Control
B7-H3 OE
B7-H3
Actin
D283 Cell lysate
B
D283
D425
D458
UW228
ONS76
HMEC
B7-H3
Actin
Cell lysates

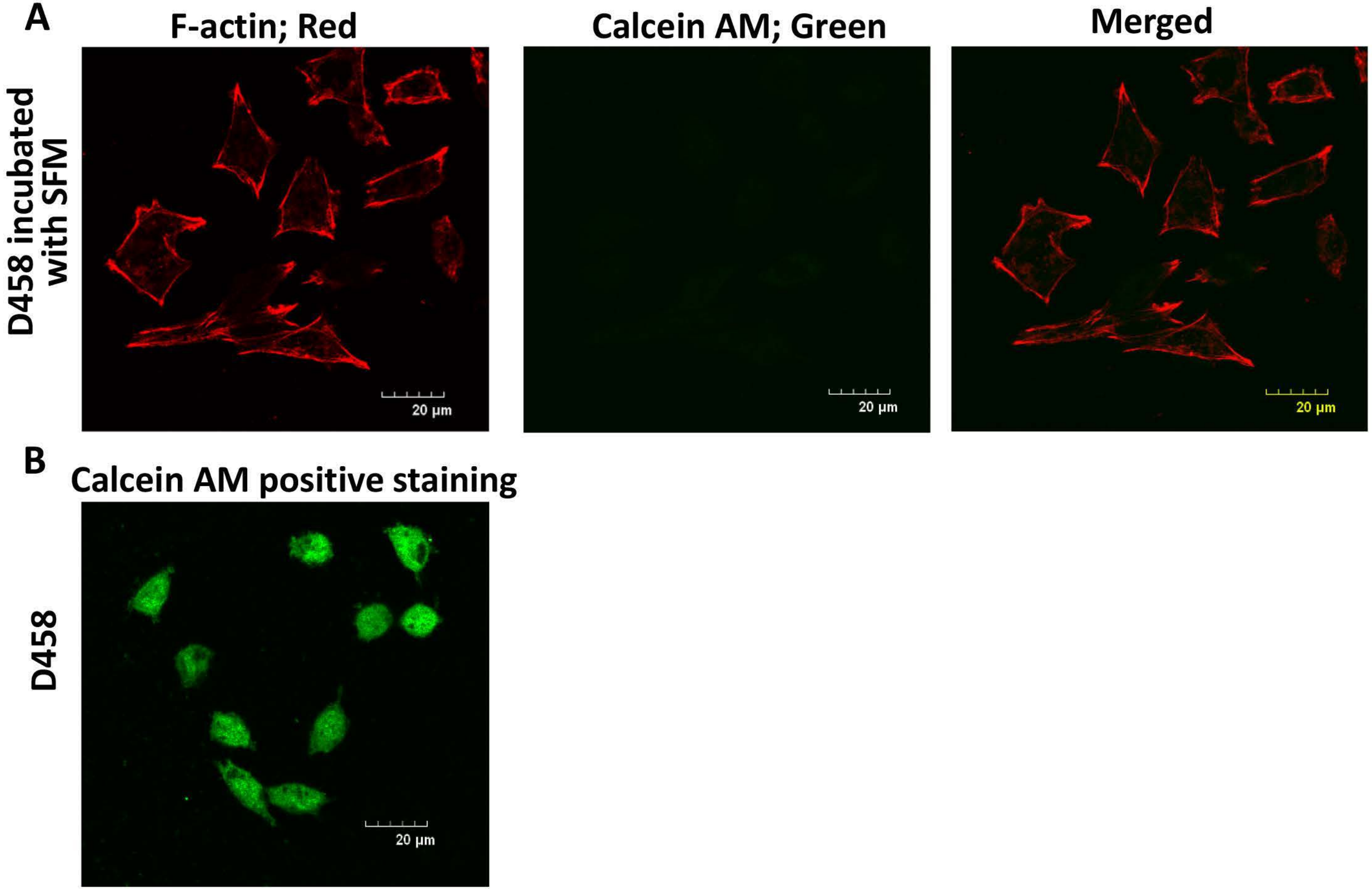
A
F-actin; Red
Calcein AM; Green
Merged
D458 incubated with SFM
20 µm
20 µm
20 µm
B
Calcein AM positive staining
D458
20 µm

Fig 3C_B7-H3 blot

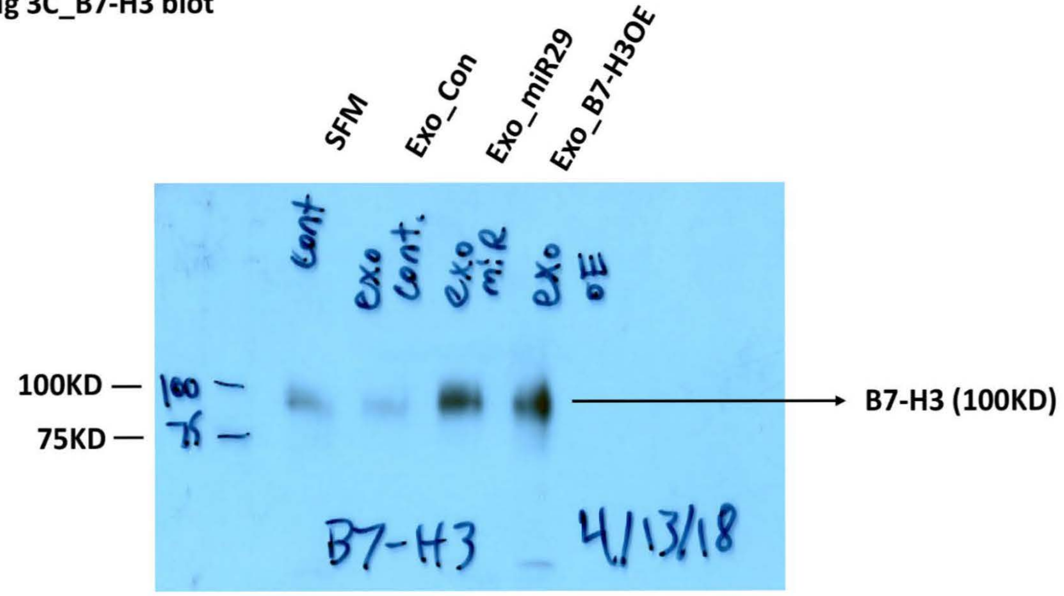

HMEC

Fig 3C_Actin blot

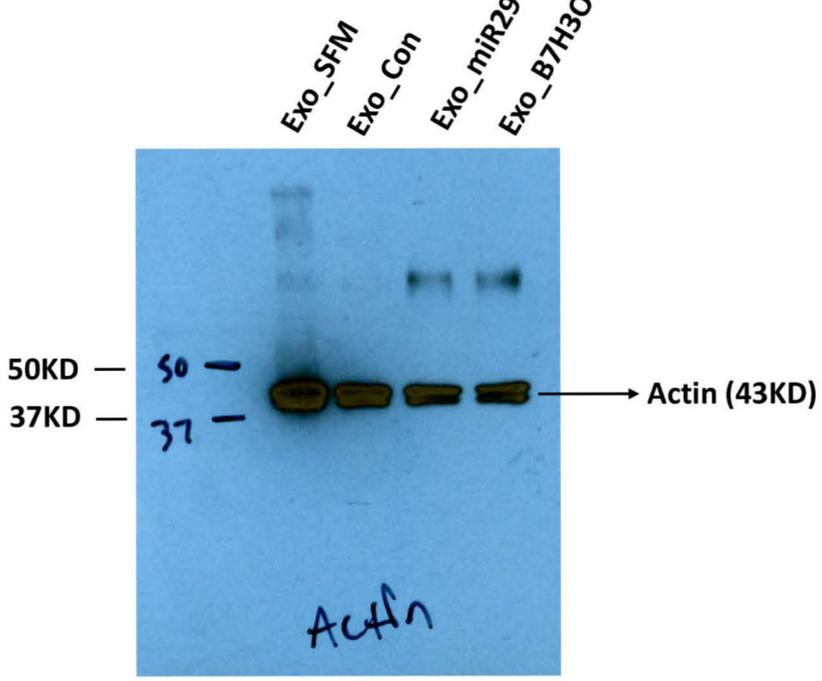

HMEC
